# Ferroptosis at the intersection of osteoarthritis and bone metabolism: mechanistic links and therapeutic prospects

**DOI:** 10.3389/fcell.2025.1722435

**Published:** 2025-12-17

**Authors:** Jing Xiao, Yan Zhu, Jingjing Chen, Yujie Hong, Yang Cao

**Affiliations:** 1 Department of Rheumatology and Immunology, Graduate School of Anhui University of Chinese Medicine, Hefei, Anhui, China; 2 Department of Rheumatology and Immunology, Second Affiliated Hospital of Anhui University of Chinese Medicine, Hefei, Anhui, China

**Keywords:** bone metabolism, chondrocytes, ferroptosis, inflammation, osteoarthritis, osteoblasts, osteoclasts

## Abstract

Osteoarthritis (OA) is a highly prevalent and debilitating degenerative joint disorder worldwide, characterized by complex pathogenesis and a lack of effective disease-modifying therapies. The traditional perspective has evolved from a simplistic “cartilage wear” model to a “whole-joint” pathology encompassing synovitis, aberrant subchondral bone remodeling, and chondrocyte death. In recent years, ferroptosis has emerged as a critical player in OA pathogenesis because of its unique regulatory mechanisms. Accumulating evidence indicates that ferroptosis contributes to OA progression through core processes, including intracellular iron overload, antioxidant system collapse, and excessive lipid peroxidation. These events not only directly trigger chondrocyte death and extracellular matrix degradation but also exacerbate bone metabolic imbalance through intricate signaling networks. Notably, the proposed “iron overload–inflammation–bone metabolism” vicious cycle underscores the central role of ferroptosis in linking cartilage degeneration to abnormal subchondral bone remodeling, providing a novel conceptual framework for understanding the “cartilage–bone” axis in OA. This review systematically outlines the molecular mechanisms of ferroptosis and its functional roles in OA chondrocytes and bone metabolism, emphasizing the pathological implications of this vicious cycle. We further discuss preclinical advances in targeting ferroptosis as a therapeutic strategy, analyze the challenges in clinical translation, and highlight future directions to inform the development of precise OA treatments.

## 1 Introduction

Osteoarthritis (OA) ranks among the leading global causes of disability and diminished quality of life, with its disease burden escalating steadily due to population aging and lifestyle changes. According to recent epidemiological data, the global prevalence of OA reached 595 million in 2020, representing an increase of over 100% since 1990, and is projected to exceed 1 billion by 2050 (GBD 2021 Osteoarthritis Collaborators, 2023). The age-standardized prevalence remains highest in high-income regions, such as North America and Europe, whereas the most rapid growth in disease burden is observed in high-income Asia-Pacific regions. This pattern likely reflects the complex interactions among socioeconomic development, demographic aging, and shifting obesity trends (GBD 2021 Osteoarthritis Collaborators, 2023). Age is the strongest non-modifiable risk factor for OA. Although the incidence peaks in the 50–64 age group, the most rapid annual increase occurs among middle-aged adults (35–49 years) (Wu et al., 2025), indicating that OA is no longer exclusively a disease of the elderly. Regarding sex differences, women face a substantially higher risk of developing OA than men. A large-scale systematic review reported a female-to-male prevalence ratio of approximately 1.7:1 (Di et al., 2024). This significant gender difference is believed to be related to the weakened protective effect of estrogen on joint cartilage and bones after menopause due to decreased estrogen levels (Li and Zheng, 2022).

The pathological understanding of OA has evolved from a “traditional cartilage-centric view” to a “whole-joint” disease concept, emphasizing the coordinated involvement of multiple articular tissues. The disease process simultaneously involves the synovium, subchondral bone, ligaments, and periarticular muscles, forming a dynamically interacting pathological network. Metabolic homeostasis in chondrocytes is critically disrupted in articular cartilage. Driven by aging, mechanical stress, or inflammation, the activities of proteases such as matrix metalloproteinases (MMPs) and ADAMTS are enhanced, accelerating the degradation of extracellular matrix (ECM) components including type II collagen and aggrecan, while simultaneously impairing their synthesis. This creates a state of “net degradation,” ultimately leading to cartilage fibrillation, fissuring, and full-threshold defects (Molitoris et al., 2024). Synovitis, once considered a secondary change, is now recognized as a key driver of OA progression. Danger signals, including cartilage degradation products, are recognized by synovial macrophages, activating pathways such as NF-κB and triggering the release of pro-inflammatory cytokines (e.g., IL-1β, TNF-α, and IL-6) and chemokines. These factors further suppress cartilage ECM synthesis and induce additional MMP production, thereby establishing a vicious cycle (Feng and Wu, 2022). Aberrant subchondral bone remodeling constitutes another critical component of OA pathology. During OA progression, bone remodeling shifts from normal homeostasis to an abnormal high-turnover state, characterized by the uncoupling of bone formation and resorption. Dysfunction of osteoclasts (OCs) and osteoblasts (OBs) represents a core mechanism underlying this dysregulation. Enhanced osteoclast activity exacerbates bone resorption (Du et al., 2023), while defective osteoblast differentiation impairs bone repair (Ren et al., 2021). The resulting imbalance between bone resorption and formation during remodeling promotes subchondral bone sclerosis and osteophyte formation (Qiao et al., 2021). Such abnormal remodeling not only compromises the mechanical support of the subchondral bone but also releases various factors that further aggravate cartilage degradation.

In recent years, ferroptosis, an iron-dependent form of regulated cell death driven by lipid peroxidation, has garnered significant attention for its pivotal role in inflammatory and degenerative diseases (Yan et al., 2021). The core mechanisms of ferroptosis involve glutathione peroxidase 4 (GPX4) inactivation, iron accumulation, and excessive peroxidation of polyunsaturated fatty acids (PUFAs), ultimately generating lethal lipid peroxides (Berndt et al., 2024; Tang et al., 2021). Recent clinical investigations have revealed significantly elevated iron concentrations in synovial fluid samples from patients with OA compared to healthy controls, with levels positively correlating with disease severity (Nugzar et al., 2018; Yao et al., 2021; Cai et al., 2021; Miao et al., 2022), suggesting the potential involvement of ferroptosis in OA pathology. In addition, a complex interplay exists between ferroptosis and bone metabolism. Early studies have demonstrated that reactive oxygen species (ROS) accumulation resulting from iron overload can simultaneously inhibit bone formation and promote bone resorption (Tsay et al., 2010). Moreover, ferroptosis in OCs disrupts bone metabolism by reducing their activity (Qu et al., 2021), indicating the critical regulatory role of ferroptosis in bone metabolism. However, the molecular mechanisms linking ferroptosis to OA-related bone metabolic imbalance remain unclear, and its precise role within the cartilage-bone microenvironment requires further investigation. Therefore, systematic investigation of the interactive mechanisms between ferroptosis and OA bone metabolism will not only contribute to refining the pathogenic theory of OA but also provide innovative targets for precision therapy, holding significant clinical translational potential.

## 2 Molecular mechanisms of ferroptosis

Ferroptosis is an iron-dependent form of regulated cell death driven by lipid peroxidation. It is distinct from apoptosis, necrosis, and autophagy, primarily characterized by the collapse of antioxidant defenses due to GPX4 inactivation and the irreversible accumulation of PUFAs (Stockwell et al., 2017). Since its initial definition by Dixon et al., in 2012, ferroptosis has emerged as a frontier in cell death research (Dixon et al., 2012).

### 2.1 Antioxidant system imbalance: collapse of ferroptosis defense

**FIGURE 1:**
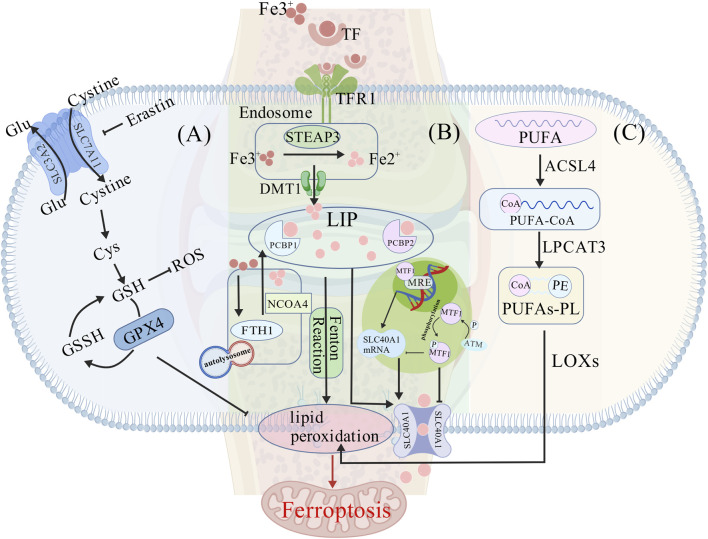
Core molecular mechanisms of ferroptosis. **(A)** Collapse of the antioxidant system. System Xc^−^, composed of SLC3A2 and SLC7A11, facilitates cystine import to sustain glutathione (GSH) synthesis. GPX4 utilizes GSH to scavenge reactive oxygen species (ROS). Inactivation of GPX4 leads to the irreversible accumulation of lipid peroxides. The ferroptosis inducer erastin inhibits system Xc^−^, depleting GSH and triggering ferroptosis. **(B)** Iron homeostasis imbalance. Transferrin receptor 1 (TFR1) mediates iron influx. Within endosomes, Fe^3+^ is reduced to Fe^2+^ by STEAP3 and released into the cytosolic labile iron pool (LIP) via divalent metal transporter 1 (DMT1). Ferritin (e.g., FTH1) iron storage is facilitated by chaperones PCBP1/2. Ferritinophagy, mediated by NCOA4, releases free Fe^2+^, which catalyzes ROS generation via the Fenton reaction. The iron efflux protein ferroportin (FPN, SLC40A1) is transcriptionally activated by MTF1 to maintain iron homeostasis. Phosphorylation of MTF1 by the ATM kinase inhibits its nuclear translocation, leading to downregulation of SLC40A1, iron overload, and consequent joint damage. **(C)** Accumulation of lipid peroxidation. Polyunsaturated fatty acids (PUFAs) are activated to PUFA-CoA by ACSL4 and then esterified into phospholipids such as phosphatidylethanolamine (PE) by LPCAT3, forming PUFA-containing phospholipids (PUFA-PLs). The peroxidation of these PUFA-PLs, catalyzed by lipoxygenases (LOXs), executes ferroptosis.

The initiation of ferroptosis is closely associated with the failure of intracellular antioxidant defense systems, with the System xc-/GSH/GPX4 axis playing a central regulatory role (Dixon et al., 2012). System xc-, a cystine/glutamate antiporter composed of SLC3A2 and SLC7A11 subunits, mediates the cellular uptake of cystine, providing the essential substrate for glutathione (GSH) synthesis (Banjac et al., 2008; Tu et al., 2021; Doll et al., 2017). GSH, a crucial antioxidant, facilitates the elimination of lipid reactive oxygen species (ROS) via GPX4. GPX4 is the unique enzyme capable of directly reducing phospholipid hydroperoxides (PLOOH); its inactivation leads to irreversible lipid ROS accumulation, thereby inducing ferroptosis (Bersuker et al., 2019; Benhar, 2020). For instance, the ferroptosis inducer erastin disrupts GSH synthesis by inhibiting SLC7A11, consequently suppressing GPX4 activity (Figure 1A). Additionally, the transcription factor Nrf2 suppresses ferroptosis by regulating the expression of GPX4 and GSH-related genes (Ezraty et al., 2017; Soeur et al., 2015; Yang et al., 2022).

### 2.2 Iron homeostasis dysregulation: free iron accumulation and ferroptosis induction

Concurrently, dysregulation of intracellular iron homeostasis represents another critical event in ferroptosis triggering (Chen X. et al., 2020; Ru et al., 2024). Increased uptake via transferrin receptor 1 (TFR1) or the release of stored iron mediated by ferritin autophagy (key regulatory factor NCOA4) can lead to the expansion of the labile iron pool (LIP) (Guo et al., 2021; Ito et al., 2021; Li JY. et al., 2024). Excess Fe^2+^ catalyzes the generation of substantial ROS through the Fenton reaction, exacerbating lipid peroxidation (Li et al., 2021). The sole cellular iron exporter, ferroportin (FPN, encoded by SLC40A1), exerts protective effects by maintaining iron homeostasis through MTF1-mediated transcriptional activation. However, ATM kinase can phosphorylate MTF1, inhibiting its nuclear translocation, downregulating SLC40A1 expression, and ultimately promoting iron overload (Montalbetti et al., 2013; Zhang et al., 2024; Xiao et al., 2023) (Figure 1B).

### 2.3 Lipid peroxidation: the executioner of ferroptosis

Ultimately, lipid peroxidation directly targets polyunsaturated fatty acid-containing phospholipids (PUFA-PLs) in cellular membranes. This process involves ACSL4 (acyl-CoA synthetase long-chain family member 4) and LPCAT3 (lysophosphatidylcholine acyltransferase 3), which mediate the esterification of PUFAs into membrane phospholipids. Subsequent peroxidation is catalyzed by lipoxygenases (LOXs) (Doll et al., 2017; Wang and Chen, 2022; Das, 2019) (Figure 1C). Under physiological conditions, the GPX4-GSH system reduces lipid peroxides, thereby suppressing ferroptosis (Koppula et al., 2021). When GPX4 activity is inhibited or GSH is depleted, accumulated lipid peroxides trigger cell death. Moreover, the protein FAF1 can sequester free PUFAs, limiting their interaction with iron and preventing lipid peroxidation (Cui et al., 2022).

In summary, lipid peroxide accumulation is the core driving force of ferroptosis. The precise regulation of iron metabolism, amino acid metabolism, and lipid metabolism collectively determines the initiation and progression of ferroptosis. Targeting these metabolic pathways may offer novel therapeutic strategies for various ferroptosis-related diseases, mitigating its detrimental effects.

## 3 Pathological features of osteoarthritis and bone metabolic imbalance

Osteoarthritis is a common chronic joint disorder characterized by cartilage degeneration, osteophyte formation, and alterations in periarticular tissues. These pathological changes interact synergistically, collectively contributing to the clinical manifestations of OA. OA was traditionally viewed as a simple “wear and tear” disorder of cartilage, but is now recognized as a metabolic and inflammatory disease involving the entire joint organ, including cartilage, subchondral bone, synovium, and ligaments. In this context, abnormal bone metabolism plays a pivotal role in OA progression.

### 3.1 Cartilage degeneration and osteophyte formation

**FIGURE 2:**
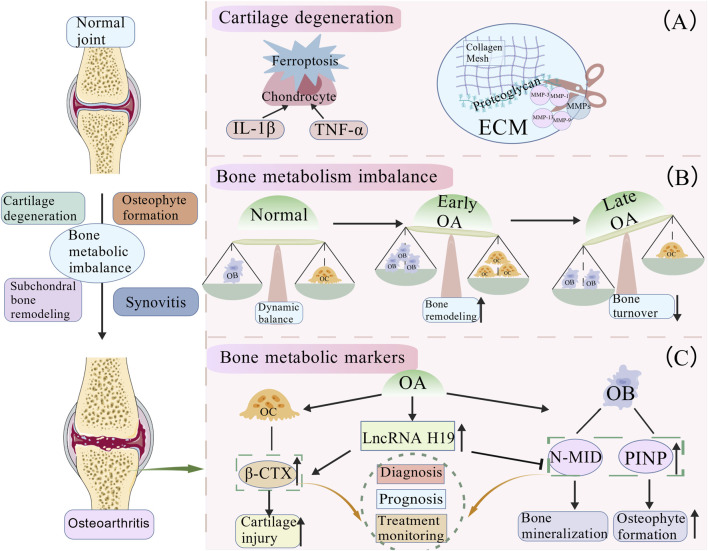
Pathological features of OA and imbalance in bone metabolism. **(A)** Cartilage degeneration. During the progression of OA from a normal joint, a series of pathological changes occur. Pro-inflammatory cytokines (e.g., IL-1β, TNF-α) and ferroptosis induce chondrocyte injury and exacerbate cartilage degradation. Aberrant activation of matrix metalloproteinases (MMPs), particularly elevated levels of MMP-1, MMP-3, MMP-9, and MMP-13, leads to the targeted degradation of type II collagen and aggrecan. This disrupts the extracellular matrix (ECM) structure and accelerates cartilage damage. **(B)** Dynamic evolution of bone metabolism during OA progression. This schematic illustrates the dynamic changes in bone metabolism from normal to late-stage OA. In the early phase, both bone resorption and formation are enhanced, leading to active bone remodeling. In the late phase, bone turnover decreases with a relative dominance of bone formation, resulting in increased bone density, trabecular thickening, and osteosclerosis. Aberrant remodeling further triggers osteophyte formation at joint margins, characterized by new bone apposition in response to increased mechanical stress. **(C)** Key regulators of bone metabolism in OA: β-CTX, PINP, and N-MID. Long non-coding RNA H19 is upregulated in OA patients. It disrupts bone metabolic balance by promoting osteoclast (OC) activity (thereby upregulating β-CTX, a marker of bone resorption) and suppressing osteoblast (OB) function (thus downregulating bone formation markers PINP and bone mineralization marker N-MID). Alterations in these biomarkers (β-CTX, PINP, N-MID) collectively reflect pathological processes in OA, such as cartilage degradation and osteophyte formation, and hold significant value for the diagnosis, prognostication, and monitoring of therapeutic responses in the disease.

Cartilage degeneration constitutes the core pathological foundation of OA, manifesting as a progressive loss of articular cartilage structure and function, leading to joint pain and functional impairment. During disease progression, chondrocytes undergo degeneration, ECM synthesis is markedly reduced, and these processes are accompanied by inflammatory cell infiltration and cytokine release, collectively accelerating cartilage damage (Aso et al., 2019). Cell death, particularly chondrocyte death, represents a critical event in OA pathology, involving multiple pathways including apoptosis and ferroptosis (Xiang et al., 2023). The mechanistic role of ferroptosis in OA and its relationship with joint tissue pathology warrant further investigation. Furthermore, MMP-mediated degradation of the cartilage matrix is a key mechanism underlying cartilage degeneration in OA. MMPs, a family of enzymes capable of degrading ECM proteins, target collagens, aggrecan, and other non-collagenous proteins (Molitoris et al., 2024). Aberrant expression and enhanced activity of MMPs during OA pathogenesis lead to structural destruction of the cartilage. Specific isoforms, including MMP-1, MMP-3, MMP-9, and MMP-13, are markedly upregulated and effectively degrade key cartilage components, such as type II collagen and aggrecan (Molitoris et al., 2024) (Figure 2A).

Subchondral bone, providing mechanical support for the articular cartilage, consists of the subchondral plate and trabecular bone, maintaining homeostasis through osteoclast-mediated bone resorption and osteoblast-mediated bone formation (Ajami et al., 2022; Li G. et al., 2013). During OA progression, the subchondral bone undergoes significant pathological changes: the early stage is characterized by active bone remodeling with increased bone resorption and formation, while the late stage shows reduced bone turnover with further diminished resorption and relatively predominant formation, leading to increased bone density, trabecular thickening, sclerosis, and subsequent osteophyte formation (Li X. et al., 2024; Donell, 2019; Hu W. et al., 2021) (Figure 2B). This aberrant remodeling not only alters the mechanical support for articular cartilage but also participates in joint metabolism and pain perception via neurovascular invasion (Hu Y. et al., 2021). Notably, pathological changes in the subchondral bone directly trigger osteophyte formation, which are bony outgrowths at joint margins considered an adaptive response to joint instability and increased mechanical stress (Aso et al., 2019). However, excessive osteophyte formation can restrict joint movement, compress surrounding neural tissues, exacerbate pain, and further impair joint function, establishing a vicious cycle.

### 3.2 Key regulators of bone metabolism in OA

Bone metabolism is a complex process involving multiple cell types and regulatory factors. The dynamic balance between osteoblasts (responsible for bone matrix synthesis and formation) and osteoclasts (mediating bone resorption and remodeling) is particularly important (Choi et al., 2024). Key biomarkers, including total procollagen type I N-terminal propeptide (T-PINP), N-terminal mid-fragment osteocalcin (N-MID), and β-C-terminal telopeptide of type I collagen (β-CTX), are closely associated with bone metabolism. 25-hydroxyvitamin D (25(OH)D), a key indicator of vitamin D status, is increasingly recognized for its regulatory role in OA pathology.

β-CTX, a degradation product of type I collagen reflecting osteoclast activity, holds significant value in OA prediction (Chen et al., 2023). Recent studies have elucidated the important role of β-CTX in OA progression from various perspectives. Multiple studies have confirmed that β-CTX (including CTX-I and CTX-II) serves as an important biomarker for OA diagnosis and disease progression monitoring (He et al., 2017). Its levels correlate with the extent of articular cartilage damage, and in knee OA, it is used alongside other markers for diagnosis and as an objective indicator for treatment response evaluation (Kothari et al., 2022; Ma et al., 2021; Xie et al., 2023; Gupte et al., 2019). In OA therapeutic research, changes in β-CTX levels reflect the efficacy of treatment. For instance, treatment with natural compounds such as Dalbergia Sissoo extract, puerarin, achyranthoside D, and galangin significantly reduced serum or urinary CTX-I and CTX-II levels, changes associated with attenuated cartilage damage, improved OARSI scores, and reduced bone degeneration (Kothari et al., 2022; Ma et al., 2021; Xie et al., 2023; Su et al., 2021). Furthermore, vitamin D influences CTX-II levels by modulating type II collagen turnover, further underscoring the importance of β-CTX in assessing cartilage metabolic status (Li et al., 2016). As a bone turnover marker, dynamic changes in β-CTX levels reflect bone metabolic processes in OA. This dynamic characteristic elucidates key aspects of OA pathology and informs the development of targeted therapeutic approaches. Consequently, β-CTX possesses substantial clinical value for the diagnosis, prognosis, and monitoring of OA. A deeper understanding of these mechanisms is crucial for developing novel therapies and improving patient outcomes.

Type I collagen, a hallmark molecule of fibrocartilage, is involved in OA pathogenesis and constitutes a major component of the bone, tendons, and synovium (Bay-Jensen et al., 2022). PINP and N-MID osteocalcin are important bone turnover markers playing significant roles in OA bone metabolism regulation. Studies have indicated that PINP levels are closely associated with OA progression. In patients with symptomatic knee OA, PINP correlates positively with bone marrow lesions, suggesting activated bone metabolism participates in early OA pathology (Ota et al., 2019). Premenopausal women with early knee OA exhibit significantly reduced serum PINP levels, indicating suppressed bone formation that is potentially linked to declining estrogen levels (Hu et al., 2022). PINP has diagnostic value for progressive knee OA: a 6-year follow-up study found that elevated PINP levels predict a higher risk of severe OA progression, particularly progressive osteophyte formation (Kumm et al., 2013). An elevated baseline PINP level indicates more severe subsequent OA progression, and persistently high levels during progression are associated with osteophyte formation (Kumm et al., 2013). Furthermore, elevated PINP levels can serve as a biomarker for rapid cartilage loss and bone-destructive OA (Arends et al., 2016). N-MID, the major form of osteocalcin synthesized by osteoblasts, reflects bone formation and mineralization status, making it a unique biomarker for studying bone metabolism in OA. Elevated expression of LncRNA H19 in the peripheral blood of patients with OA, which correlates negatively with PINP/N-MID and positively with β-CTX, implicates it in the promotion of bone resorption and inhibition of bone formation during OA development (Zhou et al., 2020). Although the molecular mechanisms of PINP and N-MID in OA have not been fully elucidated, existing evidence supports their important roles as bone turnover markers. PINP levels are associated with OA progression and serve as diagnostic and prognostic biomarkers. Concurrently, N-MID exhibits high diagnostic accuracy for OA and correlates with disease severity. Their combined application provides a comprehensive assessment of bone metabolic status in patients with OA, offering a basis for early diagnosis, disease evaluation, treatment monitoring, and prognosis judgment (Figure 2C). Future research should further explore their potential in OA pathogenesis and personalized therapies.

The association between 25(OH)D, a key vitamin D metabolite, and osteoarthritis is complex. A New Zealand cross-sectional study showed no significant correlation between serum 25(OH)D levels and chronic pain (Wu et al., 2019). In contrast, a US study reported a positive association between higher 25(OH)D levels and OA prevalence, particularly among obese individuals (Yu et al., 2023). Supporting this, an Australian cohort reported that elevated 25(OH)D levels in males were associated with an increased risk of hip replacement, a phenomenon not observed in females (Hussain et al., 2021). A systematic review integrating multiple randomized controlled trials confirmed that vitamin D supplementation offers potential benefits for pain relief and functional improvement in patients with knee OA (Wang R. et al., 2023) (Figure 2).

Osteoblasts and osteoclasts orchestrate the balance of bone remodeling through intricate signaling networks. The aberrant expression of metabolic markers, such as N-MID, PINP, β-CTX, and 25(OH)D, in OA and their correlation with disease progression suggest their potential utility as biomarkers for assessing disease severity and treatment monitoring. Future studies should elucidate the functional mechanisms of these cells and regulatory factors, validate their clinical application value, and explore their potential roles in early diagnosis and risk assessment, providing new perspectives for understanding the pathology of bone metabolism-related disorders.

## 4 The role and mechanisms of ferroptosis in OA

### 4.1 Molecular mechanisms of chondrocyte ferroptosis

Chondrocytes, the core cellular components of articular cartilage, play a vital role in maintaining joint homeostasis. Ferroptosis in chondrocytes is a key driver of cartilage degeneration, where iron overload induces cell death through lipid peroxidation, suppresses cartilage regeneration, and exacerbates OA progression (Simão and Cancela, 2021). Glutamine metabolism is essential for regulating chondrocyte function. Glutamine enters chondrocytes via the ASCT2/SLC1A5 transporter and is converted to glutamate by glutaminase (GLS), which is further metabolized into GSH by glutathione synthetase, thereby maintaining the cellular redox balance (Stegen et al., 2020; Ling et al., 2023; Lukey et al., 2013). The transcription factor SOX9 stimulates glutamine metabolism, enhances glutamine consumption and GLS1 expression, and promotes chondrocyte differentiation and function (Stegen et al., 2020). GPX4 utilizes the reducing power of GSH to eliminate lipid peroxides and ROS, thereby inhibiting ferroptosis (Stegen et al., 2020). Clinical studies have shown significantly elevated glutamine levels in the synovial fluid of patients with OA (Anderson et al., 2018; Akhbari et al., 2020). Glutamine deprivation mitigates cartilage degeneration by suppressing NF-κB activation and reducing the production of IL-1β-induced inflammatory factors (e.g., MMP3 and COX-2) and ROS (Arra et al., 2022). Furthermore, MK-801, an NMDA receptor antagonist within the glutamate signaling pathway, is capable of inhibiting the transcriptional activation of MMP3 and COX-2 by IL-1β. This observation further implies that targeting glutamate metabolism may constitute a viable strategy for delaying the progression of osteoarthritis (Piepoli et al., 2009). These findings indicate that targeting the glutamate metabolic axis to maintain the GSH/GPX4 antioxidant system may represent a novel strategy for suppressing chondrocyte ferroptosis and attenuating OA progression.

The NF-κB and MAPK signaling pathways function as master regulators of inflammation, orchestrating diverse cellular processes such as stress response, proliferation, and inflammation (Mulero et al., 2019; Guo et al., 2020; Abohassan et al., 2022; Kumar et al., 2021). Studies have shown that GPX4 deficiency activates the NF-κB/MAPK pathway, upregulating the expression of matrix-degrading enzymes such as ADAMTS5, MMP3, and MMP13, thereby exacerbating cartilage ECM degradation (Miao et al., 2022). Concurrently, GPX4 loss elevates lipid peroxidation markers (MDA and ROS), directly driving ferroptosis (Van Coillie et al., 2022). Moreover, the RNA-binding protein SND1 is aberrantly overexpressed in OA chondrocytes. It binds to HSPA5 and promotes GPX4 ubiquitination and degradation, leading to the accumulation of ROS, Fe^2+^, and MDA under IL-1β stimulation, which accelerates the ferroptosis. Knockdown of SND1 or overexpression of HSPA5/GPX4 reverses these effects (Lv et al., 2022).

**FIGURE 3:**
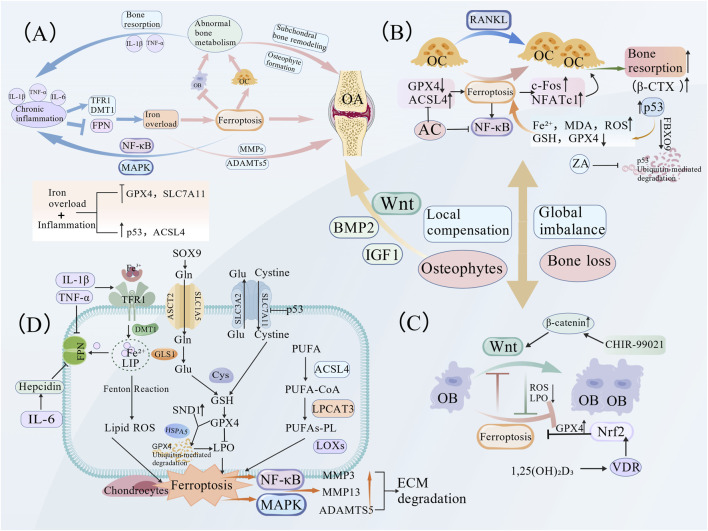
Regulatory mechanisms of ferroptosis in OA and its interplay with bone metabolism. **(A)** Model of ‘systemic imbalance with local compensation’ for ferroptosis and bone metabolism in OA. This model reveals the vicious cycle among iron overload, inflammation, and bone metabolic dysregulation. In the early stage of OA, systemic bone resorption is predominant. In the late stage, although subchondral bone and osteophyte regions exhibit a local advantage in bone formation, the overall bone metabolism remains in a negative balance. **(B)** Molecular mechanisms of osteoclast ferroptosis During RANKL-induced osteoclastogenesis, iron overload promotes osteoclast formation through iron uptake mediated by TFR1. Zoledronic acid (ZA) enhances p53 stability by inhibiting its ubiquitination and degradation mediated by FBXO9. This subsequently elevates intracellular Fe^2+^, MDA, and ROS levels while reducing GPX4 and GSH, ultimately inducing ferroptosis in osteoclasts and suppressing their activity. **(C)** Regulatory mechanisms of osteoblast ferroptosis. Iron overload suppresses osteoblast differentiation and the Wnt signaling pathway by inducing lipid peroxidation. The natural vitamin D receptor (VDR) ligand, 1,25-dihydroxyvitamin D3 (1,25(OH)_2_D3), protects osteoblasts from ferroptosis by activating the Nrf2/GPX4 pathway and reducing lipid peroxidation levels. **(D)** Role of the inflammation-iron metabolism axis in chondrocyte ferroptosis. Pro-inflammatory cytokines IL-1β and TNF-α induce intracellular iron accumulation in chondrocytes by upregulating TFR1/DMT1 and suppressing FPN expression. Iron overload, in turn, further downregulates GPX4 and SLC7A11 while activating the p53/ACSL4 axis, thereby driving lipid ROS production and ferroptosis. IL-6 exacerbates intracellular iron retention by upregulating hepcidin, which inhibits FPN.

Iron overload contributes to OA progression by linking inflammatory cytokines and iron metabolism proteins. TFR1, a type I transmembrane protein, mediates cellular iron uptake by binding transferrin (TF) and is widely expressed in the immune, hematopoietic, and neural systems (Candelaria et al., 2021; Huang et al., 2020). DMT1 specifically transports ferrous iron (Fe^2+^), and its aberrant activation promotes ROS generation via the Fenton reaction (Yanatori and Kishi, 2019). FPN, the only known iron exporter, maintains iron homeostasis by regulating iron efflux; its impairment leads to intracellular iron accumulation (Jiang et al., 2021; Li et al., 2022). Pro-inflammatory cytokines IL-1β and TNF-α upregulate TFR1 and DMT1 while suppressing FPN expression, thereby enhancing iron influx and reducing efflux in chondrocytes, resulting in iron overload (Jing et al., 2021a; Jing et al., 2021b). Iron overload downregulates GPX4 and SLC7A11 expression in chondrocytes and activates the p53/ACSL4 axis, driving lipid ROS accumulation and ferroptosis. This is accompanied by increased MMP13 and degradation of type II collagen, accelerating OA pathology (Yao et al., 2021; Ru et al., 2023; Simão et al., 2019). A key mechanistic insight is that IL-6 upregulates hepcidin, which promotes transferrin degradation and amplifies the vicious cycle of iron overload (Burton et al., 2020) (Figure 3D). These findings highlight the central role of dysregulated iron metabolism in OA, where an inflammation-driven imbalance of iron transporters exacerbates cartilage degeneration via ferroptosis. These findings support the development of OA therapies aimed at modulating cellular iron levels.

### 4.2 Association between ferroptosis and imbalance in bone metabolism

The pathological progression of OA extends far beyond the cartilage, with abnormal subchondral bone remodeling being one of its core features. Ferroptosis critically regulates OA progression by dysregulating the function of osteoblasts and osteoclasts, thereby disrupting bone homeostasis and driving subchondral bone pathology (Ru et al., 2024; Ru et al., 2023).

First, the regulation of osteoclasts by ferroptosis plays a significant role in enhanced bone resorption in OA (Xiao et al., 2025). Specifically, TfR1 upregulation promotes osteoclast differentiation and bone resorption by increasing iron uptake, directly linking cellular iron overload to elevated resorptive activity (von Brackel and Oheim, 2024). In osteoporosis, ferroptosis has been shown to participates in RANKL-induced osteoclast differentiation. The natural compound aconine (AC) inhibits NF-κB signaling (suppressing IκB and p65 phosphorylation), upregulates the key ferroptosis inhibitor GPX4, downregulates the pro-ferroptotic factor ACSL4, and significantly suppresses the expression of the core osteoclastogenic transcription factors c-Fos and NFATc1. This is accompanied by reduced serum β-CTX levels and attenuated bone resorption activity (Xue et al., 2023). Li J. et al. (2013) proposed that excess iron promotes osteoclast differentiation and bone resorption via a TNF-α-dependent mechanism, resulting in increased β-CTX levels, disrupted bone metabolism and compromised bone strength. In OA, iron overload-induced osteoclast ferroptosis may further disrupt subchondral bone metabolic homeostasis, exacerbating abnormal bone remodeling. Qu et al. (2021), in a RANKL-induced osteoclast model, found that zoledronic acid (ZA) inhibits FBXO9-mediated p53 ubiquitination and degradation, thereby enhancing p53 protein stability. This led to increased intracellular Fe^2+^, MDA, and ROS levels and decreased GPX4 and GSH levels, ultimately inducing ferroptosis and suppressing osteoclast activity (Figure 3B). These findings consistently indicate that osteoclast ferroptosis is a key mechanism regulating bone resorption. Similarly, in OA, this mechanism may contribute to pathological processes, such as aberrant subchondral bone remodeling and osteophyte formation, by amplifying the bone resorption-formation imbalance, although its deeper mechanisms require further investigation.

Osteoblasts are the primary effectors of bone formation, and their impaired function can lead to reduced bone mass and osteoporosis. Luo et al. (2022) demonstrated that iron overload induces osteoblast ferroptosis and inhibits the canonical pro-osteogenic Wnt signaling pathway. Conversely, a Wnt agonist (e.g., CHIR-99021) can suppress ferroptosis and restore osteoblast differentiation capacity by reducing ROS and lipid peroxidation levels, without altering intracellular iron concentrations. Furthermore, activating the vitamin D receptor (VDR) suppresses osteoblast ferroptosis via stimulation of the Nrf2/GPX4 pathway (Xu et al., 2022). As a natural ligand of VDR, 1,25(OH)_2_D_3_ activates Nrf2, upregulates GPX4 expression, and reduces lipid peroxidation markers (e.g., MDA and 4-HNE), thereby protecting osteoblasts from ferroptotic damage (Xu et al., 2022) (Figure 3C).

In early OA, osteoclast activity is significantly increased. Although osteoblast activity is upregulated, it is insufficient to maintain the bone resorption-formation balance, leading to alterations in the subchondral bone microstructure. Late-stage OA is characterized by a shift in bone turnover patterns, with active local bone formation in osteophyte regions accompanied by subchondral bone sclerosis (Chen et al., 2015; Ilas et al., 2018). During OA progression, ferroptosis drives aberrant subchondral bone remodeling by disrupting the dynamic balance between osteoblasts and osteoclasts. On the one hand, ferroptosis promotes osteoclast differentiation and inhibits Runx2-mediated osteoblast differentiation via the TfR1/ROS signaling axis, disrupting bone metabolic coupling and leading to subchondral bone instability, thereby exacerbating OA (Ru et al., 2023). Conversely, the initial elevation in bone resorption, as indicated by increased β-CTX (Yang M. et al., 2025) levels, combined with the late-stage suppression of bone formation by ferroptosis, results in the inhibition of overall bone metabolism. Together, these dual dysregulations form the molecular basis for dynamic changes in bone metabolic biomarkers (Ru et al., 2023). In this process, ferroptosis-mediated subchondral bone resorption releases osteoinductive factors such as insulin-like growth factor 1 (IGF1) and bone morphogenetic protein 2 (BMP2) (Qiao et al., 2021; Regan et al., 2017), stimulating compensatory osteophyte formation at the cartilage margins. Concurrently, local inflammation and mechanical stress activate the Wnt/β-catenin pathway, further promoting osteophyte development against a background of systemic impairment in osteogenic function (Chen JW. et al., 2020), thereby closely linking imbalanced bone remodeling with aberrant osteophyte formation in OA. Bone marrow mesenchymal stem cells (BMSCs) are the sources of osteoblasts and osteoclasts. Ferroptosis can further impact the activities of osteoblasts and osteoclasts by damaging these stem cells. In the pathological process of knee OA, persistent inflammation disrupts cellular iron homeostasis and downregulates SLC7A11/GPX4 by activating the MAPK pathway, inducing ferroptosis. This not only directly leads to chondrocyte death and extracellular matrix degradation but also disrupts the osteoblast-osteoclast balance by impairing BMSC function, resulting in a deteriorated subchondral bone microstructure (Li Y. et al., 2025). Furthermore, in a recent study, Li et al. confirmed through *in vivo* and *in vitro* experiments that caffeine induces osteoblast ferroptosis (evidenced by decreased GPX4 expression), thereby disrupting bone metabolic balance, causing subchondral bone loss, and ultimately aggravating OA (Li F. et al., 2025).

Studies on osteoporosis have confirmed that ferroptosis exacerbates bone loss through its dual effects on osteoblasts and osteoclasts. Iron overload-induced lipid peroxidation inhibits osteoblast differentiation while enhancing osteoclastogenesis and bone resorptive activity, driving bone remodeling towards a net resorptive state. Intriguingly, while ferroptosis contributes to high-turnover bone loss in osteoporosis, its reported effects on “bone resorption and formation” in OA studies appear contradictory, as late-stage OA is characterized by reduced bone resorption and subchondral sclerosis. This discrepancy may stem from variations in the research methodologies, OA staging criteria, and model systems. Some studies support a persistent state of bone resorption exceeding formation throughout OA progression, based on micro-CT analyses of subchondral trabeculae and marrow cavities, revealing overall bone loss and microarchitectural deterioration in patients with OA (Roberts et al., 2018; Tan et al., 2025). However, other studies emphasize localized bone formation exceeding resorption in late-stage OA, focusing on the typical pathological feature of osteophytes: research finds that although osteophyte width increases significantly in late OA, bone resorption activity still persists, but bone formation is relatively dominant (Arepati et al., 2020). These seemingly contradictory findings reflect the complexity and dynamic nature of bone turnover in OA. Therefore, bone metabolism in OA might be characterized by the “coexistence of systemic imbalance and local compensation”: systemic bone resorption predominates in the early stages, while formation exceeds resorption in osteophyte regions during the late stages; however, the overall balance may still be negative. This constitutes the core reason for the divergent conclusions across the different studies (Figure 3A).

### 4.3 A vicious cycle linking iron overload, inflammation, and bone metabolism in OA

Recent investigations have revealed that chronic, low-grade inflammation plays a pivotal role in the pathogenesis of OA. This inflammatory state disrupts the redox balance, enhances catabolic mechanisms, and induces pain. Synovial fluid from patients with OA contains abundant pro-inflammatory cytokines, including IL-1β, TNF-α, and IL-6. These key cytokines can directly disrupt the cartilage matrix structure by inducing chondrocytes to overexpress degrading enzymes, such as MMPs (Defois et al., 2022; Jenei-Lanzl et al., 2019). Consequently, the regulatory role of systemic inflammatory cytokines in chondrocytes has become a research focus. Notably, these pro-inflammatory cytokines are also dysregulated in conditions associated with iron overload. Yao et al. reported that iron overload, in conjunction with IL-1β, induces chondrocyte ferroptosis (Yao et al., 2021), thereby exacerbating the burden of OA. In experimental studies on hemophilia, iron-overloaded synovial tissues release pro-inflammatory cytokines (e.g., IL-1β and TNF-α), which significantly accelerate OA-related cartilage degenerative pathology by activating catabolic pathways in chondrocytes (Nieuwenhuizen et al., 2013).

Iron overload disrupts chondrocyte iron homeostasis, triggering OA. A study using the C-20/A4 chondrocyte cell line to simulate iron overload by increasing ferric ammonium citrate (FAC) concentration found that the treated cells exhibited increased ferritin expression but significantly decreased levels of iron regulatory proteins such as hepcidin, ferroportin, TfR1, and TfR2. An increase in intracellular labile iron leads to elevated ROS levels, reduced collagen II production, cell cycle disruption, and increased cell death (Karim et al., 2022). As discussed previously, abnormal bone metabolism contributes to OA progression, and reduced bone mass coupled with microstructural deterioration can exacerbate the disease. For instance, postmenopausal women experience increased osteoclast activity, bone loss, and elevated fracture risk owing to estrogen deficiency (McNamara, 2021). Epidemiological evidence indicates that low bone density in patients with OA is closely related to abnormal bone remodeling. A hypoxic microenvironment further contributes to a vicious cycle of bone homeostasis imbalance by inhibiting osteoblast function and promoting osteoclastogenesis (Hu et al., 2020; Usategui-Martín et al., 2022).

The interactions between iron overload, inflammation, and abnormal bone metabolism collectively constitute a vicious cycle in OA. In this model, inflammation exacerbates both ferroptosis and bone metabolic abnormalities. In turn, these abnormalities further intensify inflammation, thereby accelerating OA progression. Ferroptosis, a cell death-related process, is aggravated under iron overload conditions. Iron overload exposes articular cartilage to high iron concentrations, promoting joint degeneration with minimal potential for tissue regeneration (Simão and Cancela, 2021). The regulation of ferroptosis and intracellular iron levels is crucial for balancing cellular detoxification and inducing cell death. Abnormal bone metabolism also plays a significant role in the pathogenesis of OA. Bone metabolism is more dynamic than cartilage metabolism; however, the gradual accumulation of iron and aging may be key determinants of bone health (Simão and Cancela, 2021) (Figure 3A). Furthermore, levels of inflammatory and coagulation activation biomarkers are significantly elevated in patients with OA, indicating the involvement of multifactorial processes in OA pathogenesis (Joshi et al., 2020). In summary, the interplay between iron overload, inflammation, and abnormal bone metabolism forms a vicious cycle that drives OA progression. Understanding this cycle is crucial for developing novel therapeutic strategies (Figure 3).

## 5 Therapeutic strategies targeting ferroptosis and future research directions

Given the pivotal role of ferroptosis in OA progression, targeting its regulatory mechanisms represents a promising therapeutic strategy. In recent years, various small-molecule compounds and natural products that act on iron metabolism, lipid peroxidation, and antioxidant pathways have shown considerable therapeutic potential in experimental OA models. However, translating these findings from basic research to clinical practice presents numerous challenges. A systematic evaluation of the advantages and limitations of current therapies, along with defining future research directions, is crucial for clinical translation in this field.

### 5.1 Iron chelators: source control of iron-dependent death

Iron chelators inhibit ferroptosis at its source by sequestering excess intracellular labile iron with a high affinity. Their mechanism involves depriving the Fenton reaction of iron ions, thereby reducing ROS generation and blocking lipid peroxidation initiation. As the most extensively studied prototype, deferoxamine (DFO) exhibits clear disease-modifying efficacy, as consistently shown in OA animal models. In a primary OA model using Dunkin-Hartley guinea pigs, systemic DFO administration significantly reduced articular iron content, improved mobility, and reduced cartilage degeneration histologically (Burton et al., 2022). Further studies have indicated that DFO downregulates pro-apoptotic genes and matrix-degrading enzymes while upregulating the anti-apoptotic protein Bcl-2, suggesting multi-target protective effects. Other studies confirm that intra-articular DFO injection in murine OA models effectively reduces the expression of ferroptosis markers like GPX4 and ACSL4 in chondrocytes, inhibits IL-1β-induced accumulation of ROS and MDA, and activates the Nrf2 antioxidant pathway (Miao et al., 2022; Ma et al., 2022; Guo et al., 2023). However, the clinical translation of free DFO is hampered by its unfavorable pharmacokinetics and safety profile, including a short plasma half-life, low bioavailability, poor blood-brain barrier penetration, and potential severe adverse effects upon long-term use (Lei et al., 2024).

### 5.2 Lipid peroxidation inhibitors: directly halting membrane damage

Direct inhibition of lipid peroxidation, the biochemical hallmark and execution phase of ferroptosis, is a key therapeutic strategy. Ferrostatin-1 (Fer-1), a representative compound in this class, potently traps radicals, neutralizing lipid peroxyl radicals to halt the chain reaction of lipid peroxidation and preserve membrane integrity (Scarpellini et al., 2023). In OA models, Fer-1 effectively mitigates chondrocyte damage induced by combined inflammation and iron overload, as evidenced by enhanced cell viability, reduced lipid peroxide accumulation, activation of the SLC7A11/GPX4 pathway, and promotion of type II collagen expression (Wang D. et al., 2023). *In vivo* studies have further shown that intra-articular Fer-1 injection alleviates cartilage destruction and improves gait function in OA mice (Xu et al., 2023). Intriguingly, a comparative study of Fer-1 and another ferroptosis inhibitor, Liproxstatin-1 (Lip-1), found that while both similarly suppressed core ferroptosis markers (e.g., GPX4 and ACSL4), Fer-1 was superior in inhibiting chondrocyte catabolic markers (e.g., MMP-13 and ADAMTS-5) and restoring cartilage matrix synthesis, suggesting efficacy variations whose mechanisms warrant investigation (Yang L. et al., 2025). Despite its anti-ferroptotic potential in animal models, the clinical translation of Fer-1 is significantly limited by poor plasma stability, rapid metabolism, low bioavailability, and the lack of derivatives capable of efficiently crossing the blood-brain barrier (Yan et al., 2025). The development of novel ferroptosis inhibitors with improved pharmacokinetics and structural optimization or reformulation of existing inhibitors are critical directions for future translational research.

### 5.3 Natural products and novel compounds: a treasure trove for multi-target intervention

Natural products are an important source of ferroptosis-modulating agents. Several active components derived from traditional Chinese medicine or plants have been shown to effectively inhibit ferroptosis in OA. In IL-1β-stimulated OA chondrocytes, cardamonin (CAD) suppresses ferroptosis and the inflammatory response by modulating the p53 pathway to upregulate SLC7A11 and GPX4 expression (Gong et al., 2023). Icariin (ICA) activates the SLC7A11/GPX4 signaling pathway, mitigating chondrocyte ferroptosis and protecting cartilage (Xiao et al., 2024). Naringin (NAR) alleviates iron overload-induced cartilage damage and matrix degradation by inhibiting MMP-3/13 expression (Pan et al., 2022). This multi-target mode of action is particularly suitable for complex diseases like OA, allowing simultaneous intervention at multiple pathological nodes. However, challenges remain, including complex composition, unclear mechanisms, and limited *in vivo* efficacy of these compounds. Future research should focus on elucidating their precise molecular targets and mechanisms of action and employing medicinal chemistry approaches for structural optimization to enhance their drug-likelihood.

### 5.4 The interplay between ferroptosis and bone metabolism

The pathological features of OA extend beyond articular cartilage degeneration and include aberrant subchondral bone remodeling. Recent studies have indicated that ferroptosis plays a significant role in maintaining bone homeostasis by regulating osteoclast and osteoblast function. Elucidating this regulatory network is crucial for understanding OA pathology and developing bone remodeling-targeted therapy. Therapeutically, iron overload can inhibit osteoblast differentiation and function via ROS signaling (Wang et al., 2025). A study on osteoporosis showed that the transcription factor YAP1 directly binds to and upregulates GPX4 transcriptional activity, thereby inhibiting osteoblast ferroptosis and promoting bone formation. YAP1 deficiency exacerbates ferroptosis and bone loss (Deng et al., 2025), providing a novel target for treating bone metabolic disorders. Ishii et al. reported that TfR1-mediated iron uptake promotes osteoclastogenesis and bone resorption, which is inhibited by DFO in a dose-dependent manner (Ishii et al., 2009). A recent study by Li et al. (Li F. et al., 2025) highlighted the crucial role of gut microbiota in caffeine-associated OA progression: caffeine induces osteoblast ferroptosis, leading to subchondral bone loss, while the abundance of Prevotella copri (P. copri) decreases in coffee drinkers. The P. copri metabolite paraxanthine effectively inhibits ferroptosis. Supplementation with P. copri restores bone homeostasis and mitigates caffeine-induced subchondral bone degradation, indicating a central role for the “gut microbiota-metabolite-ferroptosis” axis in diet-related OA pathology.

### 5.5 Clinical translation challenges and strategies

Although therapeutic strategies targeting ferroptosis show great promise in preclinical research, their successful translation into clinical practice faces multiple challenges and opens up numerous avenues for future exploration. Does ferroptosis play identical roles across different stages of OA (early inflammation, middle degeneration, and late remodeling)? Do its mechanisms of action differ among various joint cell types (chondrocytes, synoviocytes, osteoblasts, and osteoclasts)? The heterogeneous pathogenesis of OA means that therapies directed against a single target often have limited efficacy. The future therapeutic paradigm is shifting towards synergistic combinations, exemplified by pairing ferroptosis inhibitors with non-steroidal anti-inflammatory drugs (NSAIDs) or chondroprotective agents, to address both symptoms and underlying pathology. Furthermore, implementing personalized precision medicine based on patient molecular profiling represents the ultimate direction for enhancing efficacy and achieving optimal cost-effectiveness of treatment.

In summary, targeting ferroptosis offers revolutionary hope for osteoarthritis. Abundant preclinical evidence, from iron chelators and lipid peroxidation inhibitors to multi-target natural products, consistently demonstrates that intervention in the ferroptosis pathway can effectively delay cartilage degeneration, suppress inflammation, and protect bone metabolism. However, translating these promising strategies from the laboratory to clinical practice remains challenging. Future research should focus on elucidating the complex mechanisms of ferroptosis and its crosstalk with other pathological processes in OA, developing precise and safe targeted delivery systems and reliable diagnostic biomarkers, and ultimately validating their efficacy and safety across diverse patient populations through rigorous clinical trials. Only then can the therapeutic potential of targeting ferroptosis be fully realized, bringing hope to hundreds of millions of patients with OA worldwide.
